# Associations between Birth Weight and Adult Sleep Characteristics: A Cross-Sectional Analysis from the UAEHFS

**DOI:** 10.3390/jcm12175618

**Published:** 2023-08-28

**Authors:** Nirmin F. Juber, Abdishakur Abdulle, Amar Ahmad, Andrea Leinberger-Jabari, Ayesha S. Al Dhaheri, Fatma Al-Maskari, Fatme AlAnouti, Mohammad Al-Houqani, Mohammed Hag Ali, Omar El-Shahawy, Scott Sherman, Syed M. Shah, Tom Loney, Youssef Idaghdour, Raghib Ali

**Affiliations:** 1 Public Health Research Center, New York University Abu Dhabi, Abu Dhabi P.O. Box 1291888, United Arab Emirates; aa192@nyu.edu (A.A.); asa12@nyu.edu (A.A.); aj100@nyu.edu (A.L.-J.); yi3@nyu.edu (Y.I.); ra107@nyu.edu (R.A.); 2 Department of Nutrition and Health, College of Medicine and Health Sciences, UAE University, Al-Ain P.O. Box 15551, United Arab Emirates; ayesha_aldhaheri@uaeu.ac.ae; 3 Institute of Public Health, College of Medicine and Health Sciences, UAE University, Al-Ain P.O. Box 15551, United Arab Emirates; fatma.am@uaeu.ac.ae (F.A.-M.); syeds@uaeu.ac.ae (S.M.S.); 4 Zayed Center for Health Sciences, UAE University, Al-Ain P.O. Box 15551, United Arab Emirates; 5 College of Natural and Health Sciences, Zayed University, Abu Dhabi P.O. Box 19282, United Arab Emirates; fatme.alanouti@zu.ac.ae; 6 Department of Medicine, College of Medicine and Health Sciences, UAE University, Al-Ain P.O. Box 15551, United Arab Emirates; alhouqani@uaeu.ac.ae; 7 Faculty of Health Sciences, Higher Colleges of Technology, Abu Dhabi P.O. Box 25026, United Arab Emirates; hagali@hct.ac.ae; 8 Department of Population Health, New York University School of Medicine, New York, NY 10016, USA; omar.elshahawy@nyulangone.org (O.E.-S.); scott.sherman@nyulangone.org (S.S.); 9 College of Medicine, Mohammed Bin Rashid University of Medicine and Health Sciences, Dubai P.O. Box 505055, United Arab Emirates; tom.loney@mbru.ac.ae; 10 MRC Epidemiology Unit, University of Cambridge, Cambridge CB2 0SL, UK

**Keywords:** birth weight, sleep characteristics, epidemiology, global health, prenatal exposure delayed effects, United Arab Emirates, UAE Healthy Future Study

## Abstract

Abnormal birth weight, particularly low birth weight (LBW), is known to have long-term adverse health consequences in adulthood, with disrupted sleep being suggested as a mediator or modifier of this link. We thus aimed to assess the associations between birth weight and self-reported adult sleep characteristics: sleep duration, difficulty waking up in the morning, daily nap frequency, sleep problems at night, snoring, daytime tiredness or sleepiness, and ever-stop breathing during sleep. This cross-sectional analysis used the United Arab Emirates Healthy Future Study data collected from February 2016 to March 2023 involving 2124 Emiratis aged 18–61 years. We performed a Poisson regression under unadjusted and age-sex-and-BMI-adjusted models to obtain the risk ratio and its 95% confidence interval for our analysis of the association between birth weight and each adult sleep characteristics, compared to individuals with normal birth weight (≥2.5 kg). Those with LBW had significantly a 17% increased risk of difficulty waking up in the morning, compared to those with normal birth weight. In addition, females with LBW history were also at an increased risk of reporting difficulty waking up in the morning. Studies with objective sleep assessments that include measurements of more confounding factors are recommended to confirm these risks.

## 1. Introduction

Good sleep is shown to fundamentally benefit human physiology and is essential for health [1,2]. In the general population, 1.6% to 18.6% of people experience severe or extreme sleep problems worldwide [3], with the prevalence reaching 56% for non-severe sleep problems [4]. Sleep disturbances can negatively affect health. However, quantifying the consequences of sleep disturbances on health is still challenging due to the lack of research involving the comprehensive domains of sleep disturbances [1]. In the general population, commonly reported sleep problems include difficulty in falling or staying asleep, difficulty waking up in the morning, and an inconsistent sleep pattern [5]. Previous epidemiological studies have documented the associations between sleep disturbances and health problems, including sleep quality, ease of falling asleep, frequent awakenings during the night, and waking up too early in the morning and psychological disorders [3,6,7], weight gain [8], and diabetes [9]. In addition, evidence suggests that sleep problems or disturbances in the population are under-reported and under-treated [4], making it harder to address these public health problems strategically.

Birth weight has been used not only as an indicator of maternal health and nutrition but also the developmental potential of the newborn [10]. Birth weight refers to the first body weight taken in the first initial hours after birth [11]. Abnormal birth weight, particularly low birth weight (LBW), is a public health concern because babies with low birth weight are known to have an increased risk of long-term adverse health consequences in adulthood compared to healthy-weight newborns [12]. LBW is defined as the birth weight of a newborn who weighs less than 2.5 kg [13], and it is predicted that 15% to 20% of newborns worldwide are LBW [14]. Previous epidemiological studies found that LBW was significantly associated with cardiometabolic disorders in adults [15,16], glucose metabolism disorders [16,17], body composition [18], and metabolic syndrome [19]. Fetal or early-life programming that occurs during embryonic and fetal development is proposed as a mechanism to explain the link between LBW and the increased risk of adult diseases in the offspring [20]. A previous study has suggested that the regulation of sleep may be programmed during early life, suggesting that LBW could have an impact on adult sleep as well [21].

Despite the fact that disrupted sleep has been suggested to mediate or modify the link between LBW and adult chronic diseases [22], there is a scarcity of population-based studies on LBW and sleep characteristics [21]. We, therefore, aimed to assess the associations between birth weight and self-reported adult sleep characteristics, namely sleep duration, difficulty waking up in the morning, daily nap frequency, sleep problems at night, snoring, daytime tiredness or sleepiness, and ever-stop breathing during sleep. We also assessed whether sex or current body mass index (BMI) modified the associations between birth weight and adult sleep characteristics.

## 2. Materials and Methods

### 2.1. Study Design, Participants, and Setting

**Figure 1:**
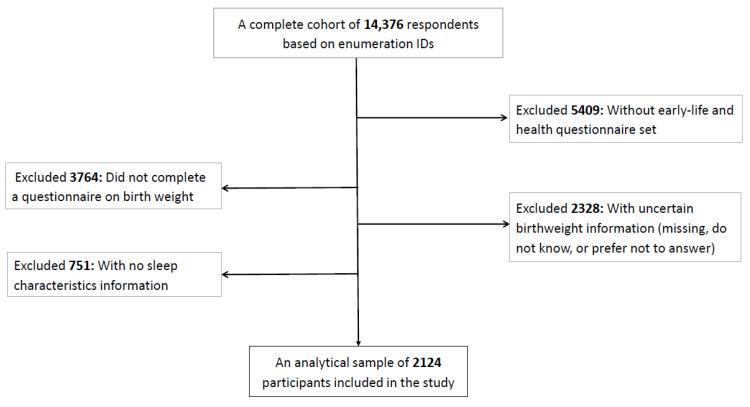
Flowchart of the final analytical sample included in the study (N = 2124).

We performed a cross-sectional study using UAE Healthy Future Study (UAEHFS) data fielded between February 2016 to March 2023 involving 2124 Emiratis aged 18–61 years who had supplied birth weight and sleep characteristics information (Figure 1). The study design, questionnaire, and methodologies of the UAEHFS are described elsewhere [23]. In brief, the UAEHFS is a population-based prospective cohort study among Emirati nationals aged 18 years and older. Emirati adults were asked to complete the questionnaire and had some physical measurements taken at multiple centers across major cities in the United Arab Emirates (UAE), namely Abu Dhabi, Al-Ain, Dubai, and Ras Al Khaimah.

### 2.2. Measurements

#### 2.2.1. Birth Weight and Adult Sleep Characteristics

Birth weight was determined based on the response to the questionnaire item: “What was your birth weight (in kg)?”, and we categorized birth weight into low birth weight (LBW) (<2.5 kg) or normal birth weight (≥2.5 kg) [24]. Less than 1% of our study participants had a reported birth weight above 4 kg (high birth weight); therefore, we labeled 2.5 kg and above as normal birth weight. A total of seven self-reported adult sleep characteristics were collected in this study. Sleep duration was determined based on the response to the questionnaire item “About how many hours sleep do you get in every 24 h including naps?”, and we categorized sleep duration into normal duration (6–9 h/day) or short/long duration (<6 or >9 h/day). Difficulty waking up in the morning was determined based on the response to the questionnaire item “On an average day, how easy do you find getting up in the morning?” (Not difficult/difficult). Daily nap frequency and sleep problems at night were determined based on the response to the respective questionnaire items “Do you have a nap during the day?” (never/rarely or sometimes/usually) and “Do you have trouble falling asleep at night or do you wake up in the middle of the night?” (never/rarely or sometimes/usually). Further, we adopted the STOP-BANG questionnaire to evaluate other adult sleep characteristics as follows [25]. Snoring was determined based on the response to the questionnaire item: “Do you snore loudly (louder than talking or loud enough to be heard through closed doors)?” (no or yes). Daytime tiredness or sleepiness was determined based on the response to the questionnaire item: “Do you often feel tired, fatigued, or sleepy during daytime?” (no or yes). Lastly, ever-stop breathing during sleep was determined based on the response to the questionnaire item: “Has anyone observed you stopping breathing during your sleep?” (no or yes).

#### 2.2.2. Demographic Characteristics

Age was constructed based on the questionnaire response to “What is your date of birth”. Sex was measured using questionnaire responses (male/female). Marital status of ever-married (divorced, separated, or widowed) and never-married (single) was classified using questionnaire responses (single/divorced/separated/widowed). Overall health status was determined based on the questionnaire response to “In general how would you rate your overall health now?” We then categorized childhood health status variables into two categories, poor or fair and good or excellent, based on the responses. Body mass index (BMI) was measured using the Tanita MC 780 (Tanita Inc., Tokyo, Japan) by nurses at the recruitment centers. We categorized BMI into normal or low (<25 kg/m^2^) and overweight or obese (≥25 kg/m^2^) [26]. Since those with a BMI <18.5 or underweight category only constituted 5% of our total participants, we combined underweight and normal weight into normal or low BMI.

### 2.3. Statistical Analysis

We evaluated demographic characteristics, birth weight, and adult sleep characteristics in the total sample, as well as among males and females, using frequencies with percentage (n, %) for categorical variables and means with standard deviations (means ± SD) for continuous variables. We fitted a Poisson regression model with robust variance to estimate the risk ratio (RR) and its 95% confidence interval (95% CI) to assess the associations between birth weight and each adult sleep characteristics under two models: unadjusted and age-sex-and-BMI-adjusted models. We adjusted for age, sex, and BMI since they are known to be strong confounding factors in epidemiological studies involving birth weight and sleep characteristics [2,6,27]. The complete case method was used to handle missing data (no data or a response of “I do not know” or “prefer not to answer”) for the regression analyses, as a previous study has shown that excluding uncertain answers in the analysis may improve the validity of health survey research [28]. Analyses were carried out using STATA 17.0 (StataCorp, College Station, TX, USA). *p* values < 0.05 were considered statistically significant.

### 2.4. Ethical Approval

The study and its procedures have been reviewed and approved by the Institutional Review Board at New York University Abu Dhabi, Dubai Health Authority, Ministry of Health and Prevention in the UAE, and Health Research and Technology Committee, reference number DOH/HQD/2020/516. Written consent was obtained from participants at the centers or by filling out an online consent form before data collection started.

## 3. Results

Table 1 shows the demographic characteristics of the sample included in the study, in the total sample, and by sex. In the total sample, we found that the mean age was 27 years, more than three-quarters resided in urban areas and reported good or excellent health, half of them were overweight or obese, and a third of them reported LBW history. By sex, compared to our male participants, females in our study had a lower mean age (25.8 vs. 28.9 years) and lower BMI (26.3 vs. 28.3 kg/m^2^), a higher proportion reported never being married (70.8% vs. 44.9%), a higher proportion had poor or fair health status (21.7% vs. 15.3%), and a higher proportion reported LBW (36.9% vs. 29.3%).

Table 2 presents the adult sleep characteristics of the sample included in the study, in the total sample, and by sex. In the total sample, we found that the majority reported normal sleep duration (70%); more than half had frequent daily naps, had sleep problems at night, and reported daytime tiredness or sleepiness; a third reported difficulty waking up in the morning; and few reported snoring (11.3%) and ever-stop breathing experience during sleep (10.6%). Compared to our male participants, more females in our study reported difficulty waking up in the morning (39.8% vs. 30%) and occasional or frequent sleep problems at night (70.4% vs. 59.2%), more females reported daytime tiredness or sleepiness (62.0% vs. 48.7%), and fewer reported snoring (7.8% vs. 15.4%) or ever-stop breathing experiences during sleep (9.0% vs. 12.5%).

Table 3 presents the risk ratios (RRs) of the associations between birth weight and adult sleep characteristics. In the crude and age-sex-and-BMI adjusted model, those with LBW history had a significantly higher risk of reporting difficulty waking up in the morning (RR = 1.20, 95% CI: 1.07–1.34 in the crude model; RR = 0.17, 95% CI: 1.04–1.33 in the adjusted model), compared to those with normal birth weight. There was no significant association observed between birth weight and other adult sleep characteristics in both crude and adjusted models.

Table 4 shows the risk ratios (RRs) of the sex-stratified associations between birth weight and adult sleep characteristics. In the crude and age-and-BMI adjusted model, females with LBW history had a significantly higher risk of reporting difficulty waking up in the morning (RR = 1.18, 95% CI: 1.03–1.36 in the crude model; RR = 1.19, 95% CI: 1.02–1.38 in the adjusted model) compared to those with normal birth weight. There was no significant association observed between birth weight and other adult sleep characteristics in either the crude or the adjusted models for males and females in our study.

Table 5 shows the risk ratios (RRs) of the BMI-stratified associations between birth weight and adult sleep characteristics. In the crude and age-sex-and-BMI adjusted model, those with normal/low BMI levels that reported LBW history had a significantly higher risk of reporting difficulty waking up in the morning (RR = 1.24, 95% CI: 1.05–1.47 in the crude model; RR = 1.20, 95% CI: 1.02–1.43 in the adjusted model), compared to those with normal birth weight. Meanwhile, in the overweight or obese group, the significant association was only observed in the crude model (RR = 1.20, 95% CI: 1.01–1.43) and diminished after adjusting for confounding factors. There was no significant association observed between birth weight and other adult sleep characteristics in either the crude or the adjusted models for participants with any BMI.

## 4. Discussion

To our knowledge, this is the first population-based study to investigate the associations between birth weight and adult sleep characteristics over a long interval. We were able to assess these long-interval associations by employing a retrospective cross-sectional design involving adults aged 18–61 years. In addition, to better understand the associations between birth weight and adult sleep characteristics and possible modification or mediation by sex or overweight/obesity in our large sample (N= 2124), we were able to stratify our analyses by sex and by BMI levels. The prevalence of LBW in our study was 33.4% (Table 1) and was shown to be higher than the previous estimates in the UAE, which revealed a LBW prevalence of 9–13% [29,30]. Our complete case analysis involving only those with certain birth weight values and complete adult sleep characteristics may explain the difference. On another note, the prevalence of sleep disturbances among our participants varied, including the average of 7 h of total sleep duration among our participants, with females having a slightly longer duration compared to males (7.1 h vs. 6.9 h) (Table 2). We have a similar finding with a previous study that revealed females in the Middle East region had 6.8 h of total sleep per night [31]. Optimally, adults need between 7–9 h of sleep per night [32]; therefore, our findings showed that Emirati adults reported normal or good sleep duration per night.

We found that participants with LBW history (<2.5 kg) had a 17% increased risk of reporting difficulty waking up in the morning compared to those with normal birthweight (≥2.5 kg), independent of age, sex, and BMI. Difficulty waking up in the morning, a phenomenon that is also called sleep inertia, is characterized by increased sleepiness, impaired cognitive or physical activity, and decreased vigilance occurring immediately after awakening from sleep [33,34]. There was evidence that showed a positive relationship between sleep inertia and its associated factors such as prior sleep deprivation and sleep problems. A previous study found that one of the critical factors for sleep inertia was the sleep stage and abrupt awakening during a slow-wave sleep episode that can lead to sleep inertia [35]. The duration of sleep inertia can be longer in some and shorter in others. Sleep inertia commonly occurs in the first 30 min post-awakening as the functional connectivity between brain networks can be strongly disrupted during this interval [34]. LBW is proposed to affect future rhythmicity through the programming of the fetal nuclei (the biological clock of the brain) and may alter sleep characteristics in adulthood. A previous study in very low birth weight (VLBW) preterm infants found that infants with VLBW had lower sleep quality and less restful night sleep compared to full-term infants [36]. Although preterm infants have several factors other than LBW that may impact their sleep architecture or circadian patterns, delayed biological maturation and adaptation among preterm infants have been suggested to affect their sleep characteristics into adulthood [37], although the exact mechanisms of how LBW history is associated with adult sleep characteristics are still unclear. Fetal or early-life programming and delayed maturation and adaptation may possibly explain the observed association between LBW history and difficulty waking up in the morning in this study. Further studies focusing on the circadian pattern among those with LBW and its association with adult sleep inertia or other sleep characteristics during adulthood are highly recommended.

We stratified our analysis of the associations between birth weight and adult sleep characteristics by sex since it has been shown that early-life programming and sleep characteristics differ by sex [6,38,39]. We found that females with LBW history, but not males with LBW history, were associated with difficulty waking up in the morning, even after adjusting for confounding factors. Low birth weight is known to be associated with hypothalamic–pituitary–adrenal (HPA) axis reactivity [38], a key mechanism linking early-life factors such as birth weight and later disease or disorders [40]. A previous systemic review of the association between early-life programming and later-life diseases suggested increased vulnerability to early-life programming in females, particularly in terms of HPA axis reactivity [38]. As an animal study further confirmed that inadequate sleep or sleep impairment may influence by HPA axis reactivity [41], we believe that females with LBW may also be more vulnerable not only to altered HPA axis reactivity due to early-life programming but also to altered sleep behavior in adulthood.

We also stratified our analysis by BMI levels, as sleep problems were shown to be associated with overweight/obesity in adults [42]. As two-thirds of Emirati adults are reported to have excess body weight [43,44], the involvement of body weight in this study is of importance. The previous significant associations of birth weight with difficulty waking up in the morning were diminished in the restricted analysis among overweight/obese adults in our study. This non-significance may suggest that overweight/obesity is a potential mediator in the association between birth weight and difficulty waking up in the morning. A previous study has revealed the association between LBW history and truncal obesity at age 50, but not with abdominal obesity [45]. Furthermore, many epidemiological studies have documented the bidirectional associations between overweight/obesity and sleep problems [27,46,47]. Normal sleep is linked to hypoventilation and overweight/obese adults have altered leptin resistance that may contribute to worsening hypoventilation [48], making them susceptible to sleep deprivation which can lead to sleep inertia. We, therefore, could argue that there is a possibility that obesity mediates the association between LBW history and adult sleep inertia. The possible mediation effect of obesity on the associations between LBW history and adult sleep characteristics in general, and specifically sleep inertia, is an interesting area for future research.

In this study, we did not find any significant associations between birth weight and other adult sleep characteristics, namely sleep duration, daily nap frequency, sleep problems at night, snoring, daytime tiredness or sleepiness, and ever-stop breathing during sleep. We could not compare our findings with another similar study since to our knowledge there has been no study of the link between birth weight and adult sleep characteristics with long intervals, similar to our study. Another study on the association between low birth weight and sleep characteristics was conducted with young adults aged 19–26 years [21]; meanwhile, our study included adults aged 18–61 years. Previous epidemiological studies reported no difference in sleep duration, sleep problems at night, or daily nap frequency among pre-term or VLBW infants vs. term or normal-weight infants by the age of 10 years or 19–26 years, respectively [21,49]. A previous study indicated that prematurity even in very preterm infants did not significantly affect sleep in the first 10 years of life and suggested that the development process of parent-to-child interaction was a primary drive for sleep behavior during childhood, rather than prematurity itself [49]. Nonetheless, we believe the direction of this research is very promising, especially with objective sleep measures, to better understand how early-life factors such as birth weight may affect adult sleep behavior or characteristics.

### Strengths and Limitations

This cross-sectional study comprehensively examined the associations between birth weight and several adult sleep characteristics, namely sleep duration, difficulty waking up in the morning, daily nap frequency, sleep problems at night, snoring, daytime tiredness or sleepiness, and ever-stop breathing during sleep. We were able to control for important confounding factors that may influence the association between birth weight and adult sleep characteristics, namely age, sex, and BMI or obesity. We were also able to maximize the representativeness of the sample by recruiting participants from multiple centers in four major cities across the UAE to improve the generalizability of this study. Despite the above strengths, our study was subject to several limitations. One limitation was self-reported birth weight and adult sleep characteristics; hence, we cannot rule out recall errors. The gold standard for birth weight is the weight of the newborn recorded by clinical staff at delivery [50]. Even though self-reported birth weight may be prone to recall bias [51], however, it has been shown to have a high degree of reliability, especially for forming birth weight categories [52]. Conversely, self-report sleep characteristics had low to moderate reliability and need to be used carefully, as more appropriate sleep measures in epidemiological population-based studies remain to be determined [53,54]. Furthermore, we adjusted for age in our basic and fully adjusted models to address the possible age effects of recall error and to improve the reliability of our study. Next, we only included those with complete information on birth weight and adult sleep characteristics; therefore, this may introduce selection bias to some degree. However, our sensitivity analyses revealed that the age and BMI of those excluded from this study due to uncertain values were similar to those included in this study (28.7 vs. 27.2 years and 27.1 vs. 27.2 kg/m^2^). Next, the sleep question of snoring adopted from the STOP-BANG questionnaire requires other people to observe the snoring [25], and those living alone could be missing the clue of snoring. Lastly, our study may be prone to residual confounding factors, such as smoking status [21]. However, maternal smoking during pregnancy is known to be an influential factor in the association between birth weight and adult sleep characteristics, and our data recorded less than 2% of maternal smoking during pregnancy.

## 5. Conclusions

Those with an LBW history, especially among females, had an increased risk of reporting difficulty waking up in the morning, which suggests sleep inertia susceptibility among those with an LBW history. A better understanding of how birth weight is linked to adult sleep characteristics will help to improve the sleep health surveillance of adults with abnormal birth weight. Studies with objective sleep assessments and that include measurement of more confounding factors are recommended to confirm these risks.

## Figures and Tables

**Table 1:** Demographic characteristics of the sample included in the study, in the total sample, and by sex (N = 2124).

Demographic Characteristics	Total Sample (N = 2124)	Male, 45.9% (N = 974)	Female, 54.1% (N = 1150)
Age, year (mean ± SD)	27.2 ± 7.6	28.9 ± 7.4	25.8 ± 7.5
Current BMI, kg/m2 (mean ± SD)	27.2 ± 6.6	28.3 ± 5.9	26.3 ± 6.9
Normal or below (<25 kg/m2)	735 (34.6)	237 (24.3)	498 (43.3)
Overweight (25 to <30 kg/m2)	554 (26.1)	319 (32.8)	235 (20.4)
Obese (≥30 kg/m2)	521 (24.5)	266 (27.3)	255 (22.2)
Missing	314 (14.8)	152 (15.6)	162 (14.1)
Marital status, n (%)			
Never married	1251 (58.9)	437 (44.9)	814 (70.8)
Ever married	873(41.1)	689 (55.1)	336 (29.2)
Urbanicity, n (%)			
Rural or other non-urban	244 (11.5)	98 (10.1)	146 (12.7)
Urban	1717 (80.8)	780 (80.1)	937 (81.5)
Missing	163 (7.7)	96 (9.9)	67 (5.8)
Education attainment, n (%)			
<6 years of schooling or missing	72 (3.4)	35 (3.6)	37 (3.2)
6 to 12 years of schooling	930 (43.8)	437 (44.9)	493 (42.9)
≥12+ years of schooling	1122 (52.8)	502 (51.5)	620 (53.9)
Overall health status, n (%)			
Poor or fair	399 (18.8)	149 (15.3)	250 (21.7)
Good or excellent	1633 (76.9)	771 (79.2)	862 (75.0)
Missing	92 (4.3)	54 (5.5)	38 (3.3)
Birth weight, n (%)			
Low birth weight or LBW (<2.5 kg)	709 (33.4)	285 (29.3)	424 (36.9)
Normal birth weight (≥2.5 kg)	1415 (66.6)	689 (70.7)	726 (63.1)

**Table 2:** Adult sleep characteristics of the sample included in the study, in the total sample, and by sex (N = 2124).

Sleep Characteristics	Total Sample (N = 2124)	Male, 45.9% (N = 974)	Female, 54.1% (N = 1150)
Sleep duration, mean ± SD	7.1 ± 2.1	6.9 ± 1.9	7.1 ± 1.9
Sleep duration, n (%)			
Normal (6–9 h/day)	1487 (70.0)	683 (70.1)	804 (69.9)
Short (<6 h/day)/Long (>9 h/day)	528 (24.9)	233 (23.9)	295 (25.7)
Missing	109 (5.1)	58 (6.0)	51 (4.4)
Difficulty waking up in the morning, n (%)			
No (easy)	1241 (58.4)	605 (62.1)	636 (55.3)
Yes (not easy)	749 (35.3)	291 (30.0)	458 (39.8)
Missing	134 (6.3)	78 (8.0)	56 (4.9)
Nap during the day, n (%)			
Never/rarely	687 (32.3)	272 (27.9)	415 (36.1)
Sometimes/usually	1328 (62.5)	639 (65.6)	689 (59.9)
Missing	109 (5.1)	63 (6.5)	46 (4.0)
Sleep problems at night, n (%)			
Never/rarely	621 (29.2)	329 (33.8)	292 (25.4)
Sometimes/usually	1387 (65.3)	577 (59.2)	810 (70.4)
Missing	116 (5.5)	68 (7.0)	48 (4.2)
Snoring, n (%)			
No	1535 (72.3)	651 (66.8)	884 (76.9)
Yes	240 (11.3)	150 (15.4)	90 (7.8)
Missing	349 (16.4)	173 (17.8)	176 (15.3)
Daytime tiredness or sleepiness, n (%)			
No	710 (33.4)	386 (39.6)	324 (28.2)
Yes	1187 (55.9)	474 (48.7)	713 (62.0)
Missing	227 (10.7)	114 (11.7)	113 (9.8)
Ever-stop breathing during sleep, n (%)			
No	1536 (72.3)	687 (70.5)	849 (73.8)
Yes	226 (10.6)	122 (12.5)	104 (9.0)
Missing	362 (17.0)	165 (17.0)	197 (17.1)

**Table 3:** Associations between birth weight and adult sleep characteristics in total sample (N = 2124).

Sleep Characteristics	Crude Model		Adjusted Model *	
	RR [95% CI]	*p*-Value	RR [95% CI]	*p*-Value
Sleep duration				
Normal birth weight	(Reference)		(Reference)	
Low birth weight	1.03 [0.88–1.20]	0.750	0.96 [0.81–1.14]	0.630
Difficulty waking up in the morning				
Normal birthweight	(Reference)		(Reference)	
Low birth weight	**1.20 [1.07–1.34]**	**0.002**	**1.17 [1.04–1.33]**	**0.012**
Nap during the day				
Normal birth weight	(Reference)		(Reference)	
Low birth weight	0.98 [0.92–1.05]	0.624	0.99 [0.92–1.06]	0.752
Sleep problems at night				
Normal birth weight	(Reference)		(Reference)	
Low birth weight	1.02 [0.96–1.09]	0.471	1.01 [0.95–1.08]	0.761
Snoring				
Normal birth weight	(Reference)		(Reference)	
Low birth weight	0.85 [0.66–1.10]	0.221	1.01 [0.77–1.31]	0.986
Daytime tiredness or sleepiness				
Normal birth weight	(Reference)		(Reference)	
Low birth weight	1.05 [0.97–1.12]	0.234	1.03 [0.95–1.11]	0.489
Ever stop breathing during sleep				
Normal birth weight	(Reference)		(Reference)	
Low birth weight	0.97 [0.75–1.26]	0.818	1.12 [0.85–1.48]	0.411

* Adjusted for age (continuous), sex (male/female), and BMI (continuous). Statistically significance at the 0.05 is marked in bold.

**Table 4:** Sex-stratified associations between birth weight and adult sleep characteristics in total sample (N = 2124).

Sleep Characteristics	Male (N = 974)				Female (N = 1150)			
	Crude Model	Adjusted Model *			Crude Model		Adjusted Model *	
	RR [95% CI]	*p*-Value	RR [95% CI]	*p*-Value	RR [95% CI]	*p*-Value	RR [95% CI]	*p*-Value
Sleep duration								
Normal birth weight	(Reference)		(Reference)		(Reference)		(Reference)	
Low birth weight	0.99 [0.77–1.26]	0.914	0.87 [0.65–1.16]	0.339	1.05 [0.86–1.28]	0.670	1.02 [0.82–1.27]	0.850
Difficulty waking up in the morning								
Normal birth weight	(Reference)		(Reference)		(Reference)		(Reference)	
Low birth weight	1.15 [0.94–1.41]	0.172	1.14 [0.92–1.43]	0.236	**1.18 [1.03–1.36]**	**0.021**	**1.19 [1.02–1.38]**	**0.024**
Nap during the day								
Normal birth weight	(Reference)		(Reference)		(Reference)		(Reference)	
Low birth weight	0.99 [0.90–1.09]	0.839	0.98 [0.88–1.08]	0.664	0.99 [0.91–1.10]	0.974	0.99 [0.90–1.10]	0.909
Sleep problems at night								
Normal birth weight	(Reference)		(Reference)		(Reference)		(Reference)	
Low birth weight	1.02 [0.91–1.13]	0.762	1.01 [0.90–1.14]	0.815	1.01 [0.93–1.08]	0.928	1.01 [0.93–1.09]	0.826
Snoring								
Normal birth weight	(Reference)		(Reference)		(Reference)		(Reference)	
Low birth weight	0.85 [0.61–1.19]	0.342	0.97 [0.69–1.37]	0.874	0.99 [0.67–1.50]	0.999	1.02 [0.66–1.57]	0.936
Daytime tiredness or sleepiness								
Normal birth weight	(Reference)		(Reference)		(Reference)		(Reference)	
Low birth weight	1.03 [0.90–1.17]	0.688	1.01 [0.88–1.17]	0.868	1.02 [0.94–1.11]	0.647	1.04 [0.95–1.13]	0.444
Ever-stop breathing during sleep								
Normal birth weight	(Reference)		(Reference)		(Reference)		(Reference)	
Low birth weight	1.05 [0.74–1.51]	0.760	1.24 [0.85–1.79]	0.261	0.93 [0.64–1.37]	0.722	0.99 [0.65–1.50]	0.959

* Adjusted for age (continuous), and BMI (continuous)**.** Statistically significance at the 0.05 is marked in bold.

**Table 5:** BMI-stratified associations between birth weight and adult sleep characteristics in total sample (N = 2124).

Sleep Characteristics	Normal/Low Weight (N = 735)				Overweight/Obese (1075)			
	Crude Model	Adjusted Model *			Crude Model		Adjusted Model *	
	RR [95% CI]	*p*-Value	RR [95% CI]	*p*-Value	RR [95% CI]	*p*-Value	RR [95% CI]	*p*-Value
Sleep duration								
Normal birth weight	(Reference)		(Reference)		(Reference)		(Reference)	
Low birth weight	0.91 [0.70–1.18]	0.465	0.90 [0.69–1.17]	0.426	1.03 [0.82–1.29]	0.829	1.01 [0.80–1.26]	0.993
Difficulty waking up in the morning								
Normal birth weight	(Reference)		(Reference)		(Reference)		(Reference)	
Low birth weight	**1.24 [1.05–1.47]**	**0.012**	**1.20 [1.02–1.43]**	**0.032**	**1.20 [1.01–1.43]**	**0.049**	1.15 [0.96–1.37]	0.141
Nap during the day								
Normal birth weight	(Reference)		(Reference)		(Reference)		(Reference)	
Low birth weight	0.99 [0.89–1.10]	0.816	0.98 [0.88–1.10]	0.751	0.97 [0.88–1.07]	0.581	0.99 [0.90–1.09]	0.830
Sleep night problems								
Normal birth weight	(Reference)		(Reference)		(Reference)		(Reference)	
Low birth weight	0.98 [0.88–1.08]	0.618	0.97 [0.88–1.07]	0.542	1.05 [0.97–1.15]	0.244	1.04 [0.96–1.14]	0.345
Snoring								
Normal birth weight	(Reference)		(Reference)		(Reference)		(Reference)	
Low birth weight	0.76 [0.41–1.40]	0.371	0.80 [0.43–1.51]	0.499	0.92 [0.68–1.25]	0.604	1.06 [0.80–1.42]	0.674
Daytime tiredness or sleepiness								
Normal birth weight	(Reference)		(Reference)		(Reference)		(Reference)	
Low birth weight	1.02 [0.91–1.14]	0.800	0.99 [0.88–1.11]	0.836	1.08 [0.98–1.20]	0.133	1.06 [0.96–1.17]	0.269
Ever-stop breathing during sleep								
Normal birth weight	(Reference)		(Reference)		(Reference)		(Reference)	
Low birth weight	1.09 [0.65–1.84]	0.742	1.18 [0.69–2.03]	0.547	1.04 [0.76–1.44]	0.799	1.11 [0.81–1.53]	0.522

* Adjusted for age (continuous), sex (male/female), and BMI (continuous)**.**

## Data Availability

The datasets used and/or analyzed during the current study are available from the senior author on reasonable request.

## References

[1] Chaput J.-P., Dutil C., Featherstone R., Ross R., Giangregorio L., Saunders T.J., Janssen I., Poitras V.J., Kho M.E., Ross-White A. (2020). Sleep timing, sleep consistency, and health in adults: A systematic review. Appl. Physiol. Nutr. Metab..

[2] Gadie A., Shafto M., Leng Y., Cam-CAN, Kievit R.A. (2017). How are age-related differences in sleep quality associated with health outcomes? An epidemiological investigation in a UK cohort of 2406 adults. BMJ Open.

[3] Koyanagi A., Stickley A. (2015). The association between sleep problems and psychotic symptoms in the general population: A global perspective. Sleep.

[4] Léger D., Poursain B., Neubauer D., Uchiyama M. (2008). An international survey of sleeping problems in the general population. Curr. Med. Res. Opin..

[5] Buboltz W., Jenkins S.M., Soper B., Woller K., Johnson P., Faes T. (2009). Sleep habits and patterns of college students: An expanded study. J. Coll. Couns..

[6] Becker S.P., Jarrett M.A., Luebbe A.M., Garner A.A., Burns G.L., Kofler M.J. (2018). Sleep in a large, multi-university sample of college students: Sleep problem prevalence, sex differences, and mental health correlates. Sleep Health.

[7] Stickley A., Leinsalu M., DeVylder J.E., Inoue Y., Koyanagi A. (2019). Sleep problems and depression among 237 023 community-dwelling adults in 46 low-and middle-income countries. Sci. Rep..

[8] Lyytikäinen P., Lallukka T., Lahelma E., Rahkonen O. (2011). Sleep problems and major weight gain: A follow-up study. Int. J. Obes..

[9] Spiegel K., Tasali E., Leproult R., Van Cauter E. (2009). Effects of poor and short sleep on glucose metabolism and obesity risk. Nat. Rev. Endocrinol..

[10] Agbozo F., Abubakari A., Der J., Jahn A. (2016). Prevalence of low birth weight, macrosomia and stillbirth and their relationship to associated maternal risk factors in Hohoe Municipality, Ghana. Midwifery.

[11] Axame W.K., Binka F.N., Kweku M. (2022). Prevalence and factors associated with low birth weight and preterm delivery in the Hohoe municipality of Ghana. Adv. Public Health.

[12] De Mendonça E.L.S.S., de Lima Macêna M., Bueno N.B., de Oliveira A.C.M., Mello C.S. (2020). Premature birth, low birth weight, small for gestational age and chronic non-communicable diseases in adult life: A systematic review with meta-analysis. Early Hum. Dev..

[13] World Health Organization (2017). The Global Health Observatory.

[14] World Health Organization (2015). Global Nutrition Targets 2025, Low Birth Weight Policy Brief 2014.

[15] Li Y., Ley S.H., VanderWeele T.J., Curhan G.C., Rich-Edwards J.W., Willett W.C., Forman J.P., Hu F.B., Qi L. (2015). Joint association between birth weight at term and later life adherence to a healthy lifestyle with risk of hypertension: A prospective cohort study. BMC Med..

[16] Yarmolinsky J., Mueller N.T., Duncan B.B., Chor D., Bensenor I.M., Griep R.H., Appel L.J., Barreto S.M., Schmidt M.I. (2016). Sex-specific associations of low birth weight with adult-onset diabetes and measures of glucose homeostasis: Brazilian Longitudinal Study of Adult Health. Sci. Rep..

[17] Jeanne T.L., Hooker E.R., Nguyen T., Messer L.C., Sacks R.M., Andrea S.B., Boone-Heinonen J. (2018). High birth weight modifies association between adolescent physical activity and cardiometabolic health in women and not men. Prev. Med..

[18] Rockenbach G., Luft V., Mueller N., Duncan B., Stein M., Vigo Á., Matos S.M., Fonseca M.J.M., Barreto S.M., Benseñor I.M. (2016). Sex-specific associations of birth weight with measures of adiposity in mid-to-late adulthood: The Brazilian Longitudinal Study of Adult Health (ELSA-Brasil). Int. J. Obes..

[19] Xiao X., Zhang Z.-X., Li W.-H., Feng K., Sun Q., Cohen H.J., Xu T., Wang H., Liu A.-M., Gong X.-M. (2010). Low birth weight is associated with components of the metabolic syndrome. Metabolism.

[20] Kwon E.J., Kim Y.J. (2017). What is fetal programming?: A lifetime health is under the control of in utero health. Obstet. Gynecol. Sci..

[21] Strang-Karlsson S., Räikkönen K., Kajantie E., Andersson S., Hovi P., Heinonen K., Pesonen A.-K., Järvenpää A.-L., Eriksson J.G., Paavonen E.J. (2008). Sleep quality in young adults with very low birth weight—The Helsinki study of very low birth weight adults. J. Pediatr. Psychol..

[22] Hovi P., Andersson S., Eriksson J.G., Järvenpää A.L., Strang-Karlsson S., Mäkitie O., Kajantie E. (2007). Glucose regulation in young adults with very low birth weight. N. Engl. J. Med..

[23] Abdulle A., Alnaeemi A., Aljunaibi A., Al Ali A., Al Saedi K., Al Zaabi E., Oumeziane N., Al Bastaki M., Al-Houqani M., Al Maskari F. (2018). The UAE healthy future study: A pilot for a prospective cohort study of 20,000 United Arab Emirates nationals. BMC Public Health.

[24] Iguacel I., Escartín L., Fernández-Alvira J.M., Iglesia I., Labayen I., Moreno L.A., Samper M.P., Rodríguez G. (2018). Early life risk factors and their cumulative effects as predictors of overweight in Spanish children. Int. J. Public Health.

[25] Alhouqani S., Al Manhali M., Al Essa A., Al-Houqani M. (2015). Evaluation of the Arabic version of STOP-Bang questionnaire as a screening tool for obstructive sleep apnea. Sleep Breath.

[26] World Health Organization (2000). Obesity: Preventing and Managing the Global Epidemic: Report of a WHO Consultation. World Health Organ Technol. Rep. Ser..

[27] Palm A., Janson C., Lindberg E. (2015). The impact of obesity and weight gain on development of sleep problems in a population-based sample. Sleep Med..

[28] Denman D.C., Baldwin A.S., Betts A.C., McQueen A., Tiro J.A. (2018). Reducing “I don’t know” responses and missing survey data: Implications for measurement. Med. Decis. Mak..

[29] The World Bank (2023). Low-Birthweight Babies—United Arab Emirates. https://data.worldbank.org/indicator/SH.STA.BRTW.ZS?locations=AE.

[30] Taha Z., Ali Hassan A., Wikkeling-Scott L., Papandreou D. (2020). Factors associated with preterm birth and low birth weight in Abu Dhabi, the United Arab Emirates. Int. J. Environ. Res. Public Health.

[31] Mindell J.A., Lee C., Sadeh A. (2017). Young child and maternal sleep in the Middle East. Sleep Med..

[32] Alafif N., Alruwaili N.W. (2023). Sleep Duration, Body Mass Index, and Dietary Behaviour among KSU Students. Nutrients.

[33] Trotti L.M. (2017). Waking up is the hardest thing I do all day: Sleep inertia and sleep drunkenness. Sleep Med. Rev..

[34] Vallat R., Meunier D., Nicolas A., Ruby P. (2019). Hard to wake up? The cerebral correlates of sleep inertia assessed using combined behavioral, EEG and fMRI measures. NeuroImage.

[35] Tassi P., Muzet A. (2000). Sleep inertia. Sleep Med. Rev..

[36] Gössel-Symank R., Grimmer I., Korte J., Siegmund R. (2004). Actigraphic monitoring of the activity-rest behavior of preterm and full-term infants at 20 months of age. Chronobiol. Int..

[37] Hoppenbrouwers T., Hodgman J.E., Rybine D., Fabrikant G., Corwin M., Crowell D., Weese-Mayer D.E. (2005). Sleep architecture in term and preterm infants beyond the neonatal period: The influence of gestational age, steroids, and ventilatory support. Sleep.

[38] Carpenter T., Grecian S., Reynolds R. (2017). Sex differences in early-life programming of the hypothalamic–pituitary–adrenal axis in humans suggest increased vulnerability in females: A systematic review. J. Dev. Orig. Health Dis..

[39] Carrier J., Semba K., Deurveilher S., Drogos L., Cyr-Cronier J., Lord C., Sekerovick Z. (2017). Sex differences in age-related changes in the sleep-wake cycle. Front. Neuroendocrinol..

[40] Reynolds R.M. (2013). Glucocorticoid excess and the developmental origins of disease: Two decades of testing the hypothesis–2012 Curt Richter Award Winner. Psychoneuroendocrinology.

[41] Van Dalfsen J.H., Markus C.R. (2018). The influence of sleep on human hypothalamic–pituitary–adrenal (HPA) axis reactivity: A systematic review. Sleep Med. Rev..

[42] Fogelholm M., Kronholm E., Kukkonen-Harjula K., Partonen T., Partinen M., Härmä M. (2007). Sleep-related disturbances and physical inactivity are independently associated with obesity in adults. Int. J. Obes..

[43] Yusufali A., Bazargani N., Muhammed K., Gabroun A., AlMazrooei A., Agrawal A., Al-Mulla A., Hajat C., Baslaib F., Philip J. (2015). Opportunistic screening for CVD risk factors: The Dubai shopping for cardiovascular risk study (DISCOVERY). Glob. Hear..

[44] Mamdouh H., Hussain H.Y., Ibrahim G.M., Alawadi F., Hassanein M., Al Zarooni A., Al Suwaidi H., Hassan A., Alsheikh-Ali A., Alnakhi W.K. (2023). Prevalence and associated risk factors of overweight and obesity among adult population in Dubai: A population-based cross-sectional survey in Dubai, the United Arab Emirates. BMJ Open.

[45] Byberg L., McKeigue P., Zethelius B., Lithell H. (2000). Birth weight and the insulin resistance syndrome: Association of low birth weight with truncal obesity and raised plasminogen activator inhibitor-1 but not with abdominal obesity or plasma lipid disturbances. Diabetologia.

[46] Fatima Y., Doi S.A., Mamun A. (2016). Sleep quality and obesity in young subjects: A meta-analysis. Obes. Rev..

[47] Patel S.R., Hayes A.L., Blackwell T., Evans D.S., Ancoli-Israel S., Wing Y.K., Stone K.L. (2014). The association between sleep patterns and obesity in older adults. Int. J. Obes..

[48] Meurling I.J., O’Shea D., Garvey J.F. (2019). Obesity and sleep: A growing concern. Curr. Opin. Pulm. Med..

[49] Iglowstein I., Latal Hajnal B., Molinari L., Largo R.H., Jenni O.G. (2006). Sleep behaviour in preterm children from birth to age 10 years: A longitudinal study. Acta Paediatr..

[50] Jaworowicz D.J., Nie J., Bonner M.R., Han D., Vito D., Hutson A., Potischman N., Trevisan M., Muti P., Freudenheim J.L. (2010). Agreement between self-reported birth weight and birth certificate weights. J. Dev. Orig. Health Dis..

[51] Tehranifar P., Liao Y., Flom J.D., Terry M.B. (2009). Validity of self-reported birth weight by adult women: Sociodemographic influences and implications for life-course studies. Am. J. Epidemiol..

[52] Nilsen T.S., Kutschke J., Brandt I., Harris J.R. (2017). Validity of self-reported birth weight: Results from a Norwegian twin sample. Twin Res. Hum. Genet..

[53] Girschik J., Fritschi L., Heyworth J., Waters F. (2012). Validation of self-reported sleep against actigraphy. J. Epidemiol..

[54] Scarlett S., Nolan H.N., Kenny R.A., O’Connell M.D. (2021). Discrepancies in self-reported and actigraphy-based sleep duration are associated with self-reported insomnia symptoms in community dwelling older adults. Sleep Health.

